# Clinical utility of RD1, RD9 and *hsp65* based PCR assay for the identification of BCG in vaccinated children

**DOI:** 10.1186/1756-0500-6-434

**Published:** 2013-10-29

**Authors:** Jeanette WP Teo, Janet WS Cheng, Roland Jureen, Raymond TP Lin

**Affiliations:** 1 Department of Laboratory Medicine, Microbiology Unit, National University Hospital, Singapore 119074, Republic of Singapore

**Keywords:** *Mycobacterium bovis* Bacille Calmette-Guérin (BCG), Adverse reaction, PCR

## Abstract

**Background:**

*Mycobacterium bovis* Bacille Calmette-Guérin (BCG) vaccine is widely administered to prevent tuberculosis. Vaccine complications are rare. However, when BCG-related adverse reactions arise there is a need to rapidly and reliably identify BCG from other members of the *Mycobacterium tuberculosis* complex (TBC). PCR assays based on the detection of the regions of difference (RD), in particular RD1 and RD9, have been invaluable in the identification of BCG. Prior to this study, specimens were identified through HPLC analysis at a local reference laboratory taking up to 2 weeks for a result. We sought to expedite the identification process by validating a RD1, RD9 and *hsp65* PCR assay for the identification and differentiation of BCG from TBC.

**Findings:**

In last past 3 years, we validated the RD1, RD9 and *hsp65* PCR assay for 16 mycobacterial isolates obtained from children who had experienced adverse reactions to BCG vaccination. In these cases, the clinician required a definitive identification of the isolate. The RD1 and RD9 PCR profiles indicated that all 16 isolates were BCG whilst amplification of the *hsp65* target functioned as a PCR positive control. When tested against clinical *M. tuberculosis* (MTB), reference and non-tuberculous mycobacteria the PCR assay demonstrated 100% sensitivity and specificity.

**Conclusions:**

The RD1, RD9 and *hsp65* PCR assay is a useful tool for the rapid and reliable identification of BCG. Its ease of use has allowed it to be implemented in our clinical microbiology laboratory.

## Findings

*Mycobacterium bovis* Bacille Calmette-Guérin (BCG) vaccine has been used extensively for almost the last 100 years for the prevention of TB. In countries with a national childhood immunization programme, vaccination rates typically exceed >80% in neonates and infants [1]. In Singapore, BCG vaccination is given to all newborns since 1957 and has contributed to an effective TB Control Programme.

The BCG vaccine is regarded as safe. There is a very low incidence of complications, ranging from 0.1 to 5 per 1000 vaccinated [2,3]. BCG-induced adverse events can be broadly classified into local or disseminated disease ranging from sub-cutaneous abscess and keloids, lymphadenopathy, suppurative lymphadenitis to systemic events such as osteitis and disseminated BCG disease [3,4]. The risk of disseminated BCG disease escalates in HIV-infected children [5]. Chemotherapy with anti-tuberculosis drugs may be initiated to prevent further progression to disseminated disease however treatment is complicated by the inherent resistance of all *M. bovis* strains to pyrazinamide [5,6]. Therefore, it is imperative to accurately identify and differentiate *M. bovis* BCG particularly from *M. tuberculosis*.

The diagnostics of BCG is not straightforward. *M. bovis* BCG belongs to the *Mycobacterium tuberculosis* complex (TBC) of highly related organisms comprising *M. tuberculosis*, *M. africanum, M. bovis, M. microti* and *M. canetii*. The lack of clear-cut differentiation between the members [7] impedes the identification of BCG. Limited methods avail for the differentiation of members of the TBC. Phenotypic biochemical assays can be highly subjective making it unreliable for identification [8]. High performance liquid chromatography (HPLC) analysis can separate BCG vaccine strains from *M. tuberculosis* and *M. bovis*[9]. However, this approach is not pragmatic for the routine diagnostic laboratory due to requirement of specialized equipment and protracted turnaround times.

Comparative genomics has revealed 16 regions of difference (RD) in *M. bovis* and *M. bovis* BCG strains, which are absent in *M. tuberculosis* H37Rv. Regions RD1, RD9, RD10 have been extensively studied. Their exclusive absence in all BCG vaccine strains and presence in TB strains makes these loci reliable and accurate diagnostic markers [10,11]. Deletion profiles based on these RD regions have been employed successfully for the differentiation of BCG and TB via PCR based approaches [12-14].

This study was initiated three years ago, in 2010, when we sought to evaluate a PCR approach for the rapid identification of BCG that would be suitable for implementation in our routine diagnostic microbiology laboratory. Prior to this, specimens were identified through HPLC analysis at a local reference laboratory. Here, we describe the clinical performance of the PCR assay based on the detection of RD1 and RD9 regions for the identification and differentiation of BCG from MTB.

Bacterial isolates used in the study comprised reference strains and clinical isolates (Table 1). Reference mycobacterial isolates were from the American Type Culture Collection (ATCC, Manassas, VA, USA) and non-mycobacterial quality control strains *Rhodococcus equi* and *Norcardia farcinica* were from our laboratory. Clinical mycobacterial specimens were obtained from (i) patients deemed to have adverse reactions to BCG immunization hence requiring definitive identification of the mycobacterial isolate (*n*=16), (ii) patients with confirmed TB (*n*=32). The diagnosis of tuberculosis was based on clinical and microbiological findings whereby the cultures were positive for *M. tuberculosis* by the Xpert MTB/RIF real-time PCR assay (Cepheid, Sunnyvale, CA) [15]. The isolation of clinical mycobacterial isolates from patient specimens is described below. Ethical approval was not required as the study was conducted for the improvement of a public health service and in a manner that subjects could not be identified.

**Table 1:** Characteristics of specimens sent for BCG identification, clinical MTB isolates and reference strains

**Samples**	**Disease description and management**	**Specimen site**	**PCR**	**result**	**PCR**	oxyR *	**SD TB Ag MPT64 Rapid test**	**AccuProbe Complex test**
			**RD1**	**RD9**	***hsp65***			
**For BCG identification (*n *** **=16)**								
Case/Sex/Age (months)								
1/M/3	BCG adentitis. Excision of inguinal lymph node	Inguinal lymph node	―	―	+	*M. bovis*	NEG	ND
2/M/2	Incision and drainage of abscess	Axillary abscess	―	―	+	*M. bovis*	NEG	POS
3/M/2	No information available	Axillary abscess	―	―	+	*M. bovis*	NEG	POS
4/F/4	Incision and drainage of gluteal abscess	Injection site abscess	―	―	+	*M. bovis*	NEG	POS
5/M/3	BCG adentitis. Excision of inguinal lymph node	Inguinal lymph node	―	―	+	*M. bovis*	NEG	POS
6/F/3	No information available	Inguinal lymph node	―	―	+	*M. bovis*	NEG	POS
7/M/3	No information available	Inguinal lymph node	―	―	+	*M. bovis*	NEG	POS
8/F/3	No information available	Inguinal lymph node	―	―	+	*M. bovis*	NEG	POS
9/M/4	BCG adenitis. Excision of inguinal lymph node	Injection site abscess	―	―	+	*M. bovis*	NEG	POS
10/M/22	No information available	Lymph node	―	―	+	*M. bovis*	NEG	POS
11/M/2	No information available	Lymph node	―	―	+	*M. bovis*	NEG	POS
12/F/3	Left caseating inguinal lymph node.	Lymph node	―	―	+	*M. bovis*	NEG	POS
13/M/3	No information available	Inguinal lymph node	―	―	+	*M. bovis*	NEG	POS
14/M/4	Left axillary lymph node abscess. Incision and drainage	Axillary abscess	―	―	+	*M. bovis*	NEG	POS
15/M/3	No information available	Lymph node abscess	―	―	+	*M. bovis*	NEG	POS
16/M/3	No information available	Inguinal lymph node aspirate	―	―	+	*M. bovis*	NEG	POS
**For specificity and sensitivity testing**								
Clinical MTB isolates (*n*= 32)		Respiratory and non-respiratory	+	+	+	ND	POS	POS
*M. tuberculosis* complex control strains (*n*=3)								
*M. bovis* BCG Pasteur ATCC 35734		NA	―	―	+	*M. bovis*	NEG	POS
*M. tuberculosis* H37Ra ATCC 25177		NA	+	+	+	MTB	POS	POS
*M. tuberculosis* H37Rv ATCC 27294		NA	+	+	+	MTB	POS	POS
Non *M. tuberculosis* strains (*n*=13)								
*M. intracellulare* ATCC 13950		NA	―	―	―	ND	ND-	ND
*M. gordonae* ATCC 35756		NA	―	―	―	ND	ND	ND
*M. kansasii* ATCC12478		NA	―	―	―	ND	ND	ND
*M. septicum* ATCC 700731		NA	―	―	―	ND	ND	ND
*M. senegalense* ATCC 35755		NA	―	―	―	ND	ND	ND
*M. perigrinum* ATCC 23001		NA	―	―	―	ND	ND	ND
*M. xenopi* ATCC 19250		NA	―	―	―	ND	ND	ND
*M. abscessus* ATCC 19977		NA	―	―	―	ND	ND	ND
*M. chelonae* ATCC 19539		NA	―	―	―	ND	ND	ND
*M. fortuitum* ATCC 6841		NA	―	―	―	ND	ND	ND
*M. haemophilum* ATCC 29548		NA	―	―	―	ND	ND	ND
*Rhodococcus equi*		NA	―	―	―	ND	ND	ND
*Norcardia farcinica*		NA	―	―	―	ND	ND	ND

ND: Not done.

NA: Not applicable.

— : No PCR amplification observed.

+ : Positive PCR amplification.

*oxyR*: A single nucleotide polymorphism, G➔A at position 285, revealed by *oxyR* sequencing differentiates *M. bovis* and TBC.

Respiratory and non-respiratory specimens (including pus and aspirate samples) were decontaminated according to standard methods using N-acetyl-L-cysteine–sodium hydroxide (NALC-NaOH) [16]. Tissue specimens were thoroughly minced using a pair of sterile scissors before the NALC-NaOH processing. Sediment from the specimen obtained by centrifugation at 3600 rpm for 15 min was resuspended in phosphate-buffered saline pH 6.8 to a final volume of 1.5 ml. Half of the sediment was used for inoculation into the automated Mycobacteria Growth Indicator Tube [MGIT, (Becton Dickinson,Cockeysville, MD)] culture system and the other half was inoculated into a Lowenstein-Jensen (LJ) slant. Cultures were incubated at 37°C for 6 weeks in MGIT and 8 weeks on LJ slants at 37°C and 5% CO_2_. Prior to transporting the pure mycobacterial cultures out of the Biosafety Level 3 Laboratory, the bacteria were killed by resuspending colonies in 500 μl of sterile water and boiled in screw cap tubes for 10 min.

The detection of two regions of difference (RD1 and RD9) was the basis of the PCR assay used to identify and differentiate BCG from TBC [13] (Table 2). The DNA template used for the PCR was 2 μl of boiled culture supernatant (above). For each isolate tested, three sets of PCR reactions were setup enabling the detection of RD1, RD9 and *hsp65* (Table 2). The amplification of a 401 bp fragment from *hsp65* served as a PCR positive control for members of the MTB complex. PCR amplification reactions were performed using HotStarTaq master mix kit (Qiagen, Hilden, Germany) with an initial denaturation at 95°C for 5 min, followed by 35 temperature cycles of heat denaturation at 94°C for 30 s, primer annealing at 62°C for 90 s and extension at 72°C for 60 s and a final step of extension at 72°C for 10 min. PCR products were separated by electrophoresis in 1.5% agarose gel, stained with ethidium bromide and visualized by UV transillumination. The results were positive when the specific size product was observed (Table 2).

**Table 2:** PCR primers used in this study

**Primer pair**	**Sequence 5’- 3’**	**Target locus**	**Amplicon size (bp)**	**Reference**
RD1 For	CCGTTGGCAGCATTGGCGGCG	RD1	126	This study
RD1 Rev	CCGGGCCCAGGAATATAGCCAG			
RD9 FF	GTGTAGGTCAGCCCCATCC	RD9	333	[13]
RD9 Int	CAATGTTTGTTGCGCTGC			
mycHsp65 left	CCGAGCTGGTCAAAGAGGTA	*hsp65*	401	This study
mycHsp65 right	CACGAAGTACCCCGAGATGT			
oxyR For	GGTGATATATCACACCATA	*oxyR*	548	[17]
oxyR Rev	CTATGCGATCAGGCGTACTTG			

Additional tests used for the identification of members of TBC include (i) AccuProbe Mycobacterium Tuberculosis Complex Culture Identification Test (Gen-Probe Incorporated, San Diego, CA) is a rapid DNA probe test utilizing nucleic acid hybridization for the identification of TBC isolated from culture. Testing was performed in accordance with the manufacturer's instructions. Briefly, after bacterial lysis, a 100 μl sample was transferred to the corresponding AccuProbe tube. Hybridization results, expressed as relative light units (RLUs), were determined with a Leader 50 luminometer (GenProbe). A positive reaction was a result > 30 000 RLU. (ii) SD TB Ag MPT64 Rapid (Standard Diagnostics, Seoul, South Korea) is an immunochromatographic test using anti-MPT64 antibodies for the detection of the MPT64 antigen of MTB isolates. The assay is used primarily to distinguish between *M. tuberculosis* complex and non-tuberculous mycobacteria (NTM). The kit was used according to the manufacturer's protocol. Briefly, 100 μl of condensation fluid from colonies growing in LJ slants were applied directly to the sample well. Tests were interpreted 15 min after sample application. The presence of only a control band alone indicated a negative result whereas the appearance of a second band (test band) indicated a positive result for MTB. (iii) *oxyR* sequencing. A single nucleotide polymorphism in position 285 of the *oxyR* sequence allows differentiation of *M. bovis* from the other members of the TBC. All *M. bovis* strains have an adenine (A) residue at nucleotide 285, whereas all *M. tuberculosis* strains have guanine (G) residue at this position [14,17]. A 548 bp fragment of *oxyR* was amplified using the primer set listed in Table 2. The PCR conditions used were based on a published PCR protocol [17]. The amplicon was purified using QIAquick PCR Purification Kit (Qiagen, Hilden, Germany) and sequenced using the same set of primers.

The administration of the BCG vaccine rarely causes complications. Mild injection site reactions are almost universal upon vaccination, often taking the form of a papule or scarring. Typically no treatment is required. Severe local and systemic BCG-induced disease is a much less frequent occurrence and may necessitate the initiation of treatment with anti-tuberculosis drugs [3,4]. Hence, it is critical to have rapid and accurate tests that can detect *M. bovis* BCG and differentiate it from MTC so that the clinician can choose the appropriate treatment.

Over the last 3 years, there were 16 cases submitted to our laboratory requesting the differentiation of BCG from MTB. BCG induced disease was suspected as adverse reactions arose shortly after vaccination and in close proximity to the injection site. Clinical descriptions of the cases where available are indicated on Table 1. In all cases with surgical intervention, the patients healed well.

Molecular assays based on the RD deletion profiles have been invaluable in differentiating members of the TBC [12-14]. The complete absence of both RD1 and RD9 is indicative of *M. bovis* BCG, conversely the presence of both RD1 and RD9 indicates *M. tuberculosis.* Non-BCG *M. bovis* is distinguished from BCG by the presence of RD1 and the absence of RD9 [12-14]. In this study, none of the 16 isolates submitted for testing were positive for RD1 or RD9, indicating they were *M. bovis* BCG. These PCR results confirmed suspicions of BCG-related disease in the recently vaccinated children (Table 1). The RD1 and RD9 PCR assay was also evaluated using clinical TB isolates (*n*=32), ATCC isolates of MTB and *M. bovis* BCG Pasteur as well as NTMs and was determined to have 100% specificity and sensitivity (Table 1). The PCR assay includes a positive amplification control designed specifically to detect the *hsp65* gene from TBC members. Performance of the *hsp65* PCR control was also excellent, exhibiting 100% specificity and sensitivity (Table 1).

*oxyR* sequencing of the 16 isolates from suspected BCG cases displayed the distinctive single nucleotide polymorphism of *M. bovis* isolates with G➔A at position 285 [17] (Table 1). The *oxyR* polymorphism however does not make a distinction between BCG and non-BCG *M. bovis*. Based on patient clinical history and the fact that zoonotic *M. bovis* infections would be almost non-existent in a non-cattle farming setting such as ours [18], *M. bovis* BCG would be the presumptive identification.

Distinguishing BCG from *M. bovis* is not a simple task. Phenotypically both species are susceptible to thiophene-2-carboxylic acid hydrazide and resistant to pyrazinamide although *M. bovis* has a preference for microaerophilic conditions compared to BCG which displays aerophilic growth (13). HPLC analysis of mycolic acid esters can be used for confirmation of BCG strains as it possesses a profile that is unique from *M. bovis* however it is a method that is restricted to being carried out at a mycobacterial reference laboratory (9). Comparatively, molecular testing offers accessibility and rapidity. Apart from the exploitation of RD profiles for species differentiation, the size-variable *senX3*-*regX3* intergenic region has also been targeted [19]. PCR assays that utilize the *senX3*-*regX3* intergenic region are also typically used in conjunction with the RD targets thereby underscoring the importance of RD for differentiating TBC members [20].

Other tests routinely performed in our laboratory for the identification of members of the TBC include the AccuProbe Mycobacterium tuberculosis complex test and the SD TB Ag MPT64 Rapid test. The AccuProbe Mycobacterium tuberculosis complex test is used for the identification of TBC members and as anticipated it did not differentiate between BCG and TB. All the BCG cases as well as the clinical TB cases tested positive with this kit (Table 1). The SD TB Ag MPT64 Rapid is an immunochromatographic test detecting MPT64, an antigen secreted by members of the TBC. Most BCG vaccine strains do not secrete MPT64 nevertheless exceptions have been noted [21,22]. Strains like BCG Tokyo and BCG Russia still retain the gene for MPT64 and the capacity to secrete the antigen [21,23]. These vaccines strains could still test positive on the SD TB Ag MPT64 Rapid test. All our MTB isolates gave positive results with the SD TB Ag MPT64 Rapid test. In contrast, all the BCG suspected cases tested negative (Table 1). In Singapore, since June 2003, BCG Danish strain 1331 has been the sole vaccine type [24]. This strain lacks MPT64 [23] and will therefore be negative on the SD TB Ag MPT64 Rapid test.

Prior to establishment of the PCR assay, specimens for BCG confirmation were sent to a reference laboratory for HPLC analysis. Here, we present evaluation data demonstrating the clinical validity of the RD1, RD9 and *hsp65* based PCR assay for the rapid detection and differentiation of *M. bovis* BCG. Its reliability and ease of use has made it feasible for incorporation as a routine mycobacterial diagnostic service in our laboratory.

This work was supported by a Health Service Development Programme Grant provided by the Ministry of Health, Singapore (Grant # HSDP06/X04).

## Competing interests

In the past 5 years none of the authors have received reimbursements, fees, funding or salary from any organization that may in any way gain or lose financially from the publication of this manuscript either now or in the future. In addition there is no non-financial competing interest to declare in relation to this manuscript.

## Authors’ contributions

JT, JC, RJ, RL conceived and participated in the study design, results analysis and the preparation of the manuscript. JT and JC performed the experiments in this study. All authors read and approved the final manuscript.
